# Rapid antigen test as a tool for the identification of SARS-CoV-2 infection and its potential as a self-testing device

**DOI:** 10.1590/0037-8682-0167-2022

**Published:** 2023-05-22

**Authors:** Priscilla Soares Filgueiras, Camila Amormino Corsini, Nathalie Bonatti Franco Almeida, Maria Luysa Camargos Pedrosa, Daniel Alvim Pena de Miranda, Sarah Vieira Contin Gomes, Jéssica Vieira de Assis, Raphael Antônio Silva, Maria Izabella Vieira de Assis Rocha Carvalho de Medeiros, Adelina Junia Lourenço, Cecilia Maria Florencio Bicalho, Raquel Virginia Rocha Vilela, Wander de Jesus Jeremias, Gabriel da Rocha Fernandes, Rafaella Fortini Grenfell e Queiroz

**Affiliations:** 1 Fundação Oswaldo Cruz, Instituto René Rachou, Diagnóstico e Terapia de Doenças Infecciosas e Câncer, Belo Horizonte, MG, Brasil.; 2 Universidade Federal de Minas Gerais, Instituto de Ciências Biológicas, Programa de Pós-Graduação em Patologia, Belo Horizonte, MG, Brasil.; 3 Universidade da Geórgia, Faculdade de Medicina Veterinária, Departamento de Doenças Infecciosas, Athens, GA, Estados Unidos da América.; 4 Fundação Benjamin Guimarães, Hospital da Baleia, Belo Horizonte, MG, Brasil.; 5 Universidade Federal de Ouro Preto, Departamento de Farmácia, Ouro Preto, MG, Brasil.; 6 Fundação Oswaldo Cruz, Instituto René Rachou, Informática de Biossistemas, Belo Horizonte, MG, Brasil.

**Keywords:** COVID-19, SARS-CoV-2, Rapid test, Antigen test, Self-test

## Abstract

**Background:**

SARS-CoV-2 virus originated in Wuhan (China) in December (2019) and quickly spread worldwide. Antigen tests are rapid diagnostic tests (RDT) that produce results in 15-30 min and are an important tool for the scale-up of COVID-19 testing. COVID-19 diagnostic tests are authorized for self-testing at home in some countries, including Brazil. Widespread COVID-19 diagnostic testing is required to guide public health policies and control the speed of transmission and economic recovery.

**Methods:**

Patients with suspected COVID-19 were recruited at the Hospital da Baleia (Belo Horizonte, Brazil). The SARS-CoV-2 antigen-detecting rapid diagnostic tests were evaluated from June 2020 to June 2021 using saliva, nasal, and nasopharyngeal swab samples from 609 patients. Patient samples were simultaneously tested using a molecular assay (RT-qPCR). Sensitivity, specificity, accuracy, and positive and negative predictive values were determined using the statistical program, MedCalc, and GraphPad Prism 8.0.

**Results:**

The antigen-detecting rapid diagnostic tests displayed 98% specificity, 60% sensitivity, 96% positive predictive value, and moderate concordance with RT-qPCR. Substantial agreement was found between the two methods for patients tested < 7 days of symptom onset.

**Conclusions:**

Our findings support the use of Ag-RDT as a valuable and safe diagnostic method. Ag-RDT was also demonstrated to be an important triage tool for suspected COVID-19 patients in emergencies. Overall, Ag-RDT is an effective strategy for reducing the spread of SARS-CoV-2 and contributing to COVID-19 control.

## INTRODUCTION

There has been a huge demand for COVID-19 self-testing worldwide owing to increased cases of the Omicron variant from the start of January 2022. Although important steps have been taken by countries to respond to the COVID-19 pandemic, all countries across all income levels remain alarmingly unprepared to meet future epidemic and pandemic threats, according to the 2021 Global Health Security Index^1^. More importantly, transmission throughout the world is still evolving^2^. On January 28, 2022, the Brazilian National Health Surveillance Agency approved the use and sale of antigen-detecting rapid diagnostic self-tests (self Ag-RDTs) for severe acute respiratory syndrome coronavirus 2 (SARS-CoV-2)^3^, following efforts by other countries, such as the United States and the United Kingdom^4^ ^,^ ^5^.

Widespread COVID-19 diagnostic testing is necessary to guide public health policies, and accelerate transmission control and economic recovery^6^ ^,^ ^7^. The real-time quantitative reverse-transcriptase polymerase chain reaction (RT-qPCR) assay is the gold standard for the diagnosis of infection^8^ ^,^ ^9^ ^,^ ^10^ ^,^ ^11^ ^,^ ^12^. However, this assay requires instrumentation and specialized knowledge. Moreover, the results of this test may take days to reach the patient, which directly influences decisions on quarantine/isolation. For cases with a "non-detectable" result, the need for isolation is more flexible, allowing the patient to return to normal activities if there are no symptoms. In cases of a "Detectable" result, the patient is advised to remain in isolation for up to 10 days after the onset of symptoms. As vaccination has been at the center of transmission control from the first peak in cases to the present date, health authorities have urged extensive testing. Self-Ag-RDTs can lead to rapid decision making on patient care, isolation, and contact tracing at the point of care^13^. This study aimed to describe the performance of Ag-RDT compared to RT-qPCR in a Brazilian cohort since self-Ag-RDT is becoming accessible. Of note, Brazil has a low testing capacity of 0.13 tests per thousand people^14^.

## METHODS

Six hundred and nine participants with suspected COVID-19 were enrolled in this study after being attended by emergency room clinicians at the Hospital da Baleia, Belo Horizonte, Brazil from June 2020 to June 2021. The nasopharyngeal samples of all participants were tested via RT-qPCR, following the recommendations of the World Health Organization (WHO)^15^. Simultaneously, swabs were rigorously collected for Ag-RDT. Samples were collected by members of the research team and the clinical staff of the hospital. For Ag-RDT analyses, participants were divided into three groups according to the collected samples: nasopharyngeal swab collection, 428; nasal swab collection, 107; and saliva collection, 74. Nasopharyngeal samples were obtained by inserting a swab through the nostril parallel to the palate. Nasal collection was performed by introducing the swab in the nasal region (i.e., the outermost part of the nose). For saliva collection, a sterile swab was inserted under the patient's tongue for approximately 5 min; thereafter, the test was performed.

The Ag-RDT test is recommended by the WHO and Brazilian Health Regulatory Agency, and is now approved as self-Ag-RDT by the Brazilian Agency (COVID Ag Detect and COVID Ag Oral Detect, ECO Diagnostica LTDA). Individual swabs were immersed in the extraction buffer provided in the kit and rotated 10 times. Three drops of the mixture were added to the sample port of the Ag-RDT, and the results were recorded after 15 min of incubation. The RT-qPCR assay was based on the detection of nucleic acids using the Allplex™ 2019-nCov Assay kit (Seegene Inc., Seoul, Republic of Korea)^16^. The detected viral targets included Gene E, Gene RdRP, and Gene N. The MS2 bacteriophage was used as an internal control. Pre-processed samples were stored at 4 °C until RNA extraction using magnetic beads. Each 25 μL reaction mixture contained 8 μL of extracted RNA, 5 μL of 2019-nCoV MOM oligonucleotides, 5 μL of nuclease-free water, 5 μL of Real-time One-step Buffer (5X), and 2 μL of Real-time One-step Enzyme (Thermo Fisher Scientific, Waltham, USA). Amplification was performed on a 7500 Fast Real-Time PCR System (Thermo Fisher Scientific, Waltham, USA), with the readings defined as Gene E - FAM, Gene RdRP - Cal Red 610, Gene N - Quasar 670, and IC - HEX. The cycling conditions were as follows: 1 cycle of 50 °C for 20 min, 1 cycle of 95 °C for 15 min, 45 cycles of 94 °C for 15 s, and 58 °C for 30 s. The amplifications were analyzed using Seegene Launcher software (Seegene Inc., Seoul, Republic of Korea).

Written informed consent was obtained from patients or their corresponding legal guardians if they were underage. This study was approved by the Ethical Human Research Committee of the Oswaldo Cruz Foundation and the National Brazilian Ethical Board (CONEP N. 30428720.3.0000.5091).

Sensitivity, specificity, accuracy, and positive and negative predictive values were calculated using MedCalc statistical software^17^. Differences were considered statistically significant when the p-value was ≤ 0.05. The agreement between methods was assessed using the Kappa Index calculated using GraphPad (GraphPad Software, Inc., San Diego, USA): k < 0.01, no agreement; k = 0.01-0.20, ‘poor’; k = 0.21-0.40, ‘fair’; k = 0.41-0.60, ‘moderate’; k = 0.61-0.80, ‘substantial’; k = 0.81-1.00, ‘almost perfect’^18^.

## RESULTS

A total of 609 participants (313 males, 59 yrs (21-97); 296 females, 53 yrs (9-97) with suspected COVID-19 were identified between June 2020 and June2021. Of these participants, 297 (48.8%) were confirmed to have COVID-19 based on positive RT-qPCR results. The median time to obtain the RT-qPCR results was 83.6 h (ranging from 24.2 to 182.3 h) while the median time to obtain the antigen test results was 15 min. At triage, the most common symptoms were dry cough (72%), dyspnea (46%), fever (37%), desaturation (20%), and myalgia (14%) (Supplementary Material 1).

Table 1 presents the diagnostic performance of the SARS-CoV-2 Ag-RDT tests compared to RT-qPCR based on GraphPad for the calculation of the Kappa Index and MedCalc statistical software for the sensitivity, specificity, accuracy, and positive and negative predictive values. Overall comparison between assays revealed a moderate degree of concordance (60% positive agreement, 98% negative agreement, 79% accuracy, and 0.58 kappa index). Two participants had invalid RT-qPCR results. Among the 607 participants with valid results, 177 (29.2%) were positive based on RT-qPCR and Ag-RDT, 303 (49.9%) were negative based on both tests, 120 (19.8%) had a positive RT-qPCR result only, and 7 (1.2%) were only positive on Ag-RDT. Discordant results were obtained for 127 (20.9%) samples, with higher discrepancy for the nasal samples 30/105 (28.6%) than the nasopharyngeal samples 81/428 (18.9%) and saliva samples 16/74 (21.6%).

**TABLE 1:** Performance of Ag-RDT using nasopharyngeal, nasal, and saliva samples compared to RT-qPCR for COVID-19 suspected participants.

	Results				Test Performance, % (95% CI)						
Parameter	TP	TN	FP	FN	Sensitivity	Specificity	PPV	NPV	Accuracy	Kappa Index	Kappa Agreement
Overall	177	303	7	120	60 (54-65)	98 (95-99)	96 (92-98)	72 (69-74)	79 (76-82)	0.58	Moderate
Age											
1-30 yrs	5	58	1	4	56 (21-86)	98 (91-100)	83 (40-97)	94 (88-97)	93 (84-98)	0.63	Substantial
31-60 yrs	88	120	2	62	59 (50-67)	98 (94-100)	98 (92-99)	66 (62-70)	77 (71-81)	0.55	Moderate
61-80 yrs	64	98	3	44	59 (49-69)	97 (92-99)	96 (87-99)	69 (64-74)	78 (71-83)	0.56	Moderate
>81 yrs	20	27	1	10	67 (47-83)	96 (82-100)	95 (74-99)	73 (62-82)	81 (69-90)	0.62	Substantial
**Number of days with symptoms**											
<3	9	37	0	8	53 (28-77)	100 (91-100)	100	82 (74-89)	85 (73-93)	0.61	Substantial
4-7	112	213	5	68	62 (55-69)	98 (95-99)	96 (90-98)	76 (72-79)	82 (78-85)	0.62	Substantial
8-10	54	46	2	42	58 (45-70)	94 (79-99)	95 (83-99)	52 (44-59)	70 (59-79)	0.43	Moderate
>11	2	7	0	2	53 (36-70)	100 (86-100)	100	59 (50-67)	72 (59-83)	0.47	Moderate
**Type of sample**											
Nasopharyngeal	124	223	4	77	62 (5-68)	98 (96-100)	97 (96-100)	74 (71-78)	81 (77-85)	0.61	Substantial
Nasal	29	46	2	28	51 (37-64)	96 (86-100)	94 (79-98)	62 (56-68)	71 (62-80)	0.45	Moderate
Saliva	24	34	1	15	66 (45-77)	97 (85-100)	96 (77-99)	69 (60-77)	78 (67-87)	0.58	Moderate

**TP:** true positive; **TN:** true negative; **FP:** false positive; **FN:** false negative; **CI:** confidence interval; **PPV:** positive predictive value; **NPV:** negative predictive value.

Differences were observed when groups were separated by age, number of days with symptoms, and samples collected. Of the 609 participants tested, 297 (48.7%) were positive based on RT-qPCR and 185 (30.4%) were tested using Ag-RDT. Among them, nasopharyngeal, nasal, and saliva samples were collected from 128, 32, and 25 cases, respectively, for the Ag-RDT tests. A comparison of the methods revealed substantial agreement when participants were diagnosed within the first 8 days of symptoms, despite higher sensitivity when diagnosis was performed between days 4 and 7 of symptoms. Nasopharyngeal samples led to better concordance between methods (substantial agreement, 0.61 kappa index). Further, the use of saliva for Ag-RDT led to a higher sensitivity than the use of other samples. 

## DISCUSSION

In the ongoing context of the COVID-19 pandemic, diagnostic testing is critical for limiting the spread of the virus and managing infected participants during isolation. In the first months of the pandemic, considerable challenges were made regarding the use of nucleic acid tests and clinical characteristics as reference standards for a definitive diagnosis of patients^19^. Many advances have been made, and RT-qPCR has become a reliable detection method of when performed on days 3 to 7 of symptom onset^20^. Despite its sensitivity, RT-qPCR is a time-consuming method with several practical issues, including the need for specialized operators and certified laboratories. As a result, the use of RT-qPCR is particularly challenging in resource-limited settings. The above information is particularly relevant to the reality of developing countries that face budget constraints and are below the ideal level in relation to their mass testing capacity^21^. Accordingly, it is imperative that tests are available as public policies adopted by health authorities. Based on the evidence, successful strategies implemented worldwide include aggressive testing and isolation^22^ ^,^ ^23^. Testing capacity is inversely associated with the mortality rate, which is another indication of the impact of the effective tracing of positive participants^24^.

Despite all efforts, diagnostic reports take days to become available, thereby having an excessive impact on the hospital’s operating costs due to the unnecessary isolation and management of symptomatic participants. Costs during acute COVID-19 infection are considered to be substantially higher than those for other common infectious diseases, such as influenza and pertussis (4-5.5 times higher)^25^ ^,^ ^26^ ^,^ ^27^ ^,^ ^28^. A study conducted in a hospital in São Paulo, Brazil revealed an average cost of approximately US$12,637.42 for hospitalization due to COVID-19, which is almost double that of other countries^29^. The direct medical costs are higher as a patient with COVID-19 has a greater probability of hospitalization and mortality than individuals infected with other pathogens. Additionally, positive individuals require follow-up care and potential rehospitalization due to long-lasting damage, with considerable medical costs remaining after acute infection^30^.

In our study, the time to obtain the RT-qPCR results ranged from 24.2 to 182.3 h (median 83.6 h); however, the Ag-RDT results were obtained in only 15 min. The sensitivity and accuracy of Ag-RDT were 60% and 79%, respectively, with accuracy higher on the first seven days of symptom onset (< 3, 85%; 4-7, 82%) and substantial agreement with the results of the reference, RT-qPCR. The length of SARS-CoV-2 RNA present in the upper and lower respiratory tracts and extrapulmonary specimens remains undetermined^31^. Further, RT-qPCR sensitivity decreases from >90% on days 1 to 3 post-symptoms to nearly 80% on day 6 and <50% by day 14^32^. However, a study revealed persistent detection of SARS-CoV-2 RNA in 23% of nasopharyngeal and oropharyngeal swab samples for up to 3 weeks after the first positive result, and up to 4 weeks of detection in 14% of patients^33^. More supportive data are needed to gain a clear understanding of the kinetics of viral loads based on antigen levels, despite a strong correlation between antigen levels and the viral load during the clinical course, with a similar declining trend after 8 days of symptoms^34^. As RT-qPCR can identify viral fragments, a prolonged positive result does not always indicate an active particle; therefore, these individuals are not infectious^35^. 

While other researchers found that older age is correlated with higher viral load^34^, a slight difference in diagnosis was found in this study when individuals were divided by age. Notably, other demographics and clinical characteristics did not differ between participants who tested positive or negative for SARS-CoV-2 RNA^36^ ^,^ ^37^, as revealed herein.

‘Moderate’ agreement was found between Ag-RDT and RT-qPCR (κ = 0.58), an agreement that was ‘Substantial’ on the first seven days of symptoms. Testing may be initially negative in participants, especially those who will later develop overt COVID-19, and is not surprising considering the kinetics of SARS-CoV-2 infection. The incubation period for SARS-CoV-2 is believed to extend to 14 days, with a median time of 4-5 days from exposure to symptomatic onset^30^. Transmission might be possible for approximately 8 days after symptom appearance. The period from the first day of detection to virus clearance is usually 12 days in symptomatic patients^37^ ^,^ ^38^ ^,^ ^39^. Although the significance of transmission remains unclear, virus shedding in some participants may continue for some days after symptom relief^40^ ^,^ ^41^. Other researchers have questioned the low performance of antigen detection as frontline testing, but have not considered the testing time^10^ ^,^ ^36^, which was confirmed to be imperative for both RNA and antigen detection in this study. 

Ag-RDT reproduced the RT-qPCR results for 78.7% of participants and did not show any inconclusive results, demonstrating a strong colorimetric reaction for positive samples and complete absence of color for negative samples. RT-qPCR revealed two inconclusive results (0.33% of cases). Of these inconclusive cases, one was positive based on Ag-RDT on day 7 of dyspnea, with pulmonary multifocal ground-glass opacities on both sides on chest tomography. Among the seven participants positive based on Ag-RDT between days 4 and 10 of symptoms, all presented symptoms suggesting COVID-19, such as fever, dry cough, and dyspnea, as well as chest tomography alterations. RT-qPCR plays a crucial role in accurately detecting SARS-CoV-2 on a case-by-case basis; however, it also has inherent problems that limit its utility. False-negative and invalid results may occur due to mutations in the primer and probe target regions in the SARS-CoV-2 genome, even when based on the conserved regions of the viral genomes^31^. Demographic factors, such as age, sex, and time of collection, may also play a role, especially as false-negative results are associated with the detection threshold of the test^42^. These differences may be related to the difference found in the performance of the test provided by the manufacturer, as the sensitivity of the test for samples from the Brazilian Nasopharyngeal was 88.7% (81.3-93.4%)^43^. RT-qPCR may also lead to inconclusive results due to low viral load in the very early or late phase of the disease, mutation of the virus, or other technical difficulties in handling samples^44^.

Management and isolation start with diagnosing patients with suspected COVID-19. Although the intensity of the symptoms is being reduced and a greater number of people with mild symptoms are being reported, society cannot be restricted to diagnosis in health units. The entire population must be prepared for rapid decisions regarding isolation when positive and the transmission of SARS-CoV-2 remains high. A low threshold should be set for suspicion and confirmation of infection. Efforts should be made to conduct testing and management in rapidly accessible areas with a low risk of exposure to restrict contact with viruses to reduce transmission^44^ ^,^ ^45^ ^,^ ^46^, especially in countries with a low testing capacity. Ag-RDT tests can markedly expand access and speed of testing and have a greater impact on public health than laboratory-based molecular methods. The data presented here show the similarity of test performance using nasopharyngeal and saliva samples, which may be related to recent findings showing a higher level of ACE2 expression in salivary glands than in the lungs^47^. A study of 200 patients in Bangkok revealed the sensitivity of detection of SARS-CoV-2 viral RNA in 84% of samples compared to molecular detection using nasopharyngeal and throat samples^48^. Other studies reinforce these findings regarding the detection of viral antigens in saliva samples, showing high viral load in samples from oropharyngeal health care workers^49^. 

The collection of saliva is quite simple and associated with little hassle, and may help decrease the risk of infection at the time of collection. Saliva has been used for the detection of other respiratory viruses, highlighting its remarkable utility^40^. Of note, this study reports the results obtained from symptomatic patients; the performance of these tests in asymptomatic patients cannot be evaluated^50^. Furthermore, the presence of viral particles in saliva during SARS-CoV-2 infection is a key part of the viral shedding process, as saliva droplets can be expelled during coughing or even speaking. Therefore, control measures were used, such as the use of masks, to prevent viral spread in this study. Ag self-RDT may be utilized locally, avoiding the need for centralized testing facilities that rarely meet the needs of participants, especially in low- and middle-income countries^51^.

Our findings revealed that the Ag-RDT test is an easy-to-perform diagnostic platform and is developing into a safe approach for distinguishing symptomatic contagious individuals with SARS-CoV-2 from non-contagious individuals. In addition, for developing countries where the population has limited access to diagnostic facilities, the Ag-RDT test is a necessary tool that enables effective decision-making and consequently, stricter control of transmission.

## Appendix

**SUPPLEMENTARY MATERIAL 1:** Demographic data and characteristics of 609 patients admitted to Hospital da Baleia, Belo Horizonte from June 2020 to June 2021 with suspected COVID-19; detectable and non-detectable RT-qPCR.

	RT-qPCR	
Age (years)	Detected	Not detected
	N	
<20	5	19
21-30	7	13
31-40	19	20
41-50	49	42
51-60	77	59
61-70	68	64
71-80	40	38
>81	28	25
**Symptoms**	**N**	
Dyspnoea	146	157
Dry cough	137	155
Fever	136	108
Desaturation	80	60
Myalgia	57	33
Diarrhea	46	13
Hyporexy	41	19
Headache	35	24
Emesis or nausea	28	28
Odinophagy	23	15
Tachypnea	21	21
Anosmia or dysgeusia	20	8
Runny nose	19	32
Productive cough	13	11
Asthenia	12	10
Mental confusion	10	9
Fatigue	7	2
Hypoxemia	5	4
Convulsion	1	4
**Medical Severity**	**N**	
Ward	228	234
ICU	73	74
**Patient outcome**	**N**	
Hospital discharge	241	227
Death	71	72
